# Simultaneous determination of zircon U–Pb age and titanium concentration using LA-ICP-MS: Analysis data and crystallization temperature

**DOI:** 10.1016/j.dib.2020.106092

**Published:** 2020-07-29

**Authors:** Takashi Yuguchi, Kozue Ishibashi, Shuhei Sakata, Tatsunori Yokoyama, Daichi Itoh, Yasuhiro Ogita, Koshi Yagi, Takeshi Ohno

**Affiliations:** a Faculty of Science, Yamagata University, 1-4-12 Kojirakawa, Yamagata 990-8560, Japan; b Earthquake Research Institute, University of Tokyo, 1-1-1 Yayoi, Bunkyo-ku, Tokyo 113-0032, Japan; c Japan Atomic Energy Agency, 959-31, Jorinji, Izumi-cho, Toki, Gifu 509-5102, Japan; d Hiruzen Institute for Geology and Chronology Co., Ltd., 2-5, Nakashima, Naka-ku, Okayama 703-8252, Japan; e Faculty of Science, Gakushuin University, 1-5-1 Mejiro, Toshima-ku, Tokyo, 171-8588 Japan

**Keywords:** Zircon U–Pb age and titanium concentration, Crystallization age and temperature, Time–temperature (*t–T*) path, LA-ICP-MS, Granitic rock, Biotite K–Ar age

## Abstract

Simultaneous determination of zircon U–Pb age and titanium concentration for a single analysis spot gives both the crystallization age and temperature. In laser ablation inductively coupled plasma mass spectrometry (LA-ICP-MS) analysis, it is challenging to quantitatively analyse a low level of titanium concentration. Two approaches were employed using a quadrupole mass spectrometer equipped with a collision/reaction cell (CRC). In the first approach, the MS/MS mass-shift mode with oxygen reaction gas provided reliable and consistent measurement of titanium as ^48^Ti^16^O^+^. In the second approach, the titanium concentration was determined quantitatively from the signal intensity of ^49^Ti in the non-gas mode (without the inflow of collision/reaction gas into the CRC). The methods were applied to zircon samples of the Kurobegawa granite (KRG), the Okueyama granite (OKG), the Toki granite (TKG), and the Tono plutonic complex (TCP). The biotite K–Ar geochronology were employed for rock samples of the KRG, OKG, and TPC (N = 3) of which the zircon crystals were analysed. The obtained titanium concentrations of the zircon crystals can lead to the crystallization temperatures through Ti-in-zircon geothermometer.

Specifications Table

**Table:** 

Subject	Geochemistry and Petrology
Specific subject area	Zircon geochronology
Type of data	Table
How data were acquired	ICP-mass spectrometry coupled with laser ablation sample introduction technique (LA-ICP-MS)
Data format	Raw and analysed
Parameters for data collection	Simultaneous determination of zircon U–Pb age and titanium concentration
Description of data collection	In the first system using Agilent 8800 triple quadrupole ICP-MS, MS/MS mass-shift mode with oxygen reaction gas provides the reliable and consistent measurement of titanium as a ^48^Ti^16^O^+^. In the second system using Agilent 7700 quadrupole ICP-MS, the titanium concentration is determined quantitatively from the signal intensity of ^49^Ti without the inflow of reaction gas into the CRC.
Data source location	The Kurobegawa granite (KRG), the Okueyama granite (OKG), the Toki granite (TKG), and the Tono plutonic complex (TCP)
Data accessibility	With the article
Related research article	Authors’ names: Takashi Yuguchi, Kozue Ishibashi, Shuhei Sakata, Tatsunori Yokoyama, Daichi Itoh, Yasuhiro Ogita, Koshi Yagi, and Takeshi Ohno Title: Simultaneous determination of zircon U–Pb age and titanium concentration using LA-ICP-MS for crystallization age and temperature. Journal: Lithos DOI: https://doi.org/10.1016/j.lithos.2020.105682

**Value of the Data**

• U–Pb age and titanium concentration of standard zircon using the LA-ICP-MS will use as reference data to assess the age and temperature data obtained from the unknown samples in future zircon study.
• The data contribute to the petrologist, mineralogist and geochronologist using the LA-ICP-MS in the long term because standard zircon data will be employed as reference data and granitic zircon data will be used as a consideration of cooling and exhumation history in local geology.
• The data derived from two system display different error and lower limit of quantification for each system, which will be used for a consideration of analysis condition in future experiments.

## 1 Data Description

Simultaneous determination of zircon U–Pb age and titanium concentration for a single analysis spot was obtained using the ICP-mass spectrometry coupled with laser ablation sample introduction technique (LA-ICP-MS). In the LA-ICP-MS (α) system using Agilent 8800 triple quadrupole ICP-MS, MS/MS mass-shift mode with oxygen reaction gas provides the reliable and consistent measurement of titanium as a ^48^Ti^16^O^+^. In the LA-ICP-MS (β) system using Agilent 7700 quadrupole ICP-MS, the titanium concentration is determined quantitatively from the signal intensity of ^49^Ti without the inflow of reaction gas into the collision/reaction cell (CRC).

U-Pb isotopic data of the GJ-1 and Plešovice zircons and titanium concentrations of the 91500 zircons (Table S1) and U-Pb isotopic and concentration data of the KRG and OKG (Table S2) were obtained from the LA-ICP-MS (α) system. U-Pb isotopic data of the Temora 2 and OD-3 zircons and titanium concentrations of the 91500 zircons (Table S3) and U-Pb isotopic and concentration data of the KRG, TKG and TPC (Table S4) were obtained from the LA-ICP-MS (β) system. The rock samples of the KRG, OKG, and TPC were dated by biotite K-Ar geochronology. Table S5 consists of potassium, radiogenic argon concentrations, and the isotopic ages of the samples. Temperature estimation of zircon crystallization deduced from the Ti-in-zircon thermometer of Ferry and Watson [1] (Table S6).

## 2 Experimental Design, Materials and Methods

In the simultaneous determination of zircon U–Pb age and titanium concentration, it is challenging to quantitatively analyse a low level of titanium concentration using LA-ICP-MS analysis. A quadrupole mass spectrometer equipped with a collision/reaction cell (CRC) can effectively remove the ion, which interferes with the detection of the target ion, through 1) the principle of kinetic energy discrimination (KED) (collision mode) and 2) the difference in chemical reactivity between the target ion and reactant gas (e.g., hydrogen, oxygen, and methane), and the interfering ion and reactant gas (reaction mode). Accurately determining ^48^Ti is enabled by the usage of a quadrupole mass spectrometer equipped with CRC. In fact, titanium determination monitoring the signal intensity of ^48^Ti suppresses the counting error because of the high isotope abundance ratio of ^48^Ti, which yields high signal intensity in the ICP-MS analysis. Therefore, Two approaches were employed for determining the quantitative titanium concentration: determination based on 1) signal intensity of ^48^Ti using the reaction mode in the ICP mass spectrometer equipped with CRC as the LA-ICP-MS (α) system and 2) the signal intensity of ^49^Ti without collision/reaction gas as the LA-ICP-MS (β) system. The simultaneous determination of zircon U–Pb age and titanium concentration was applied to zircon samples from the Kurobegawa granite (KRG), Okueyama granite (OKG), Toki granite (TKG), and Tono plutonic complex (TPC). The crystallization temperature could then be estimated using Ti-in-zircon thermometry (Ferry and Watson [1]).

## 3 Ethics Statement

Hereby, I consciously assure that for the manuscript:

1) This material is the authors' own original work, which has not been previously published elsewhere.
2) The paper reflects the authors' own research and analysis in a truthful and complete manner.
3) The paper properly credits the meaningful contributions of co-authors.
4) All authors have been personally and actively involved in substantial work leading to the paper, and will take public responsibility for its content.

I agree with the above statements and declare that this submission follows the policies of Lithos and Elsevier as outlined in the Guide for Authors and in the Ethics Statement.

Date: 14^th^ July 2020

Corresponding author's signature: Takashi YUGUCHI

## Declaration of Competing Interest

This research was carried out under a contract with the Ministry of Economy, Trade and Industry (METI) as part of its R&D supporting program titled “Establishment of Advanced Technology for Evaluating the Long-term Geosphere Stability (2019 Fy)” A part of this work was financially supported by a JSPS KAKENHI for Young Scientists [grant number 16H06138] to TY.

The authors declare that they have no known competing financial interests or personal relationships which have, or could be perceived to have, influenced the work reported in this article.

## Appendix Supplementary materials

Image, application 1
